# Average Force of Deployment and Maximum Arrest Force of Energy Absorbers Lanyards

**DOI:** 10.3390/ijerph17207647

**Published:** 2020-10-20

**Authors:** Elena Ángela Carrión, Pedro Ignacio Saez, Juan Carlos Pomares, Antonio Gonzalez

**Affiliations:** 1 Building & Urban Development Department, University of Alicante, 03690 Alicante, Spain; pi.saez@ua.es; 2 CRESMES, Research Group for Testing, Simulation and Modelling Structures in Civil Engineering and Architecture, University of Alicante, 03690 Alicante, Spain; 3 Civil Engineering Department, University of Alicante, 03690 Alicante, Spain; jc.pomares@ua.es (J.C.P.); antonio.gonzalez@ua.es (A.G.)

**Keywords:** energy absorber lanyard, fall arrest systems, dynamic performance test, arrest force

## Abstract

Energy-absorbing lanyards (EAL) are part of fall arrest systems (FAS), their main mission is to dissipate the energy generated during the fall, ensuring that the arresting force does not cause injury to the user. For the design of FAS as set out in the American standard Z359.6 and the Canadian Z259.16 it is essential to know the deployment force or average arrest force (Fa). Fa is necessary to estimate the elongation that the absorber will suffer during the fall and therefore essential data to calculate the clearance distance. There is a lack of useful experimental data for the design of this personal protective equipment (PPE). This work provides empirical data required for the design of FAS with EAL in accordance with EN 355. This paper covers different types of EAL that are marketed internationally; different empirical data, average and maximum forces, required for improving safety design are researched. Six manufacturers, 10 models, and 2 samples of each model were selected, with total of 20 tests being performed. Dynamic performance tests were carried out, the free fall of a person was simulated using a 100 kg steel ballast from the maximum height allowed by the equipment, obtaining the maximum arrest force (Fm), average deployment force (Fa), and, by calculating the balance of forces, the maximum and average acceleration suffered by the ballast during its arrest. In light of the results, relevant conclusions for user safety are obtained. It is feasible to raise the safety requirements established by the different standards. The Fm can be established below 6 kN in the EAL, and the Fa can be estimated at 87.5% of the Fm. The categorization of the force–time curve in fall arrest with EAL has been obtained. Two EAL purchased on the market exceed the Fm permitted, therefore it is recommended to increase the quality controls of EAL.

## 1. Introduction

Accidents due to falls from a height are the cause of the highest number of fatalities in the construction industry over the last decade, and it is observed that these accidents are growing at higher rates than the number of workers in the sector [1,2,3]. This negative trend persists, as can be seen in the study of accidents occurring in the United States from 1997 to 2012, where accidents due to falls from a height increased from 36% in the Huang study in 2003 to approximately 45% in 2017 [4]. Personal protect equipment (PPE), in particular fall arrest systems (FAS), is important to the safety of construction workers [5], and we have the moral responsibility to continue studying, improving, and contributing to continuous improvement in the field of preventing falls from a height.

In Australia [6], the fall-from-height fatality rate was 13% in 2018. In the building construction and construction services, falls from a height is the first cause of mortality, representing 12% and 20% of mortality, respectively. In Singapore [7], the highest number of deaths occurred in the construction industry; falls from heights have been the greatest cause of fatal injuries at 32%. Of serious accidents, 10% are due to falls from a height, only surpassed by falls at the same level and traffic accidents. In Spain [8], statistics from the Ministry of Employment and Social Security for the year 2018 reveal that falls from a height represent 12% of all fatal accidents. A recent study [9] shows that in 7.3% of the fatal falls in the construction industry the FAS was in use by the workers, and obviously it failed.

**Figure 1:**
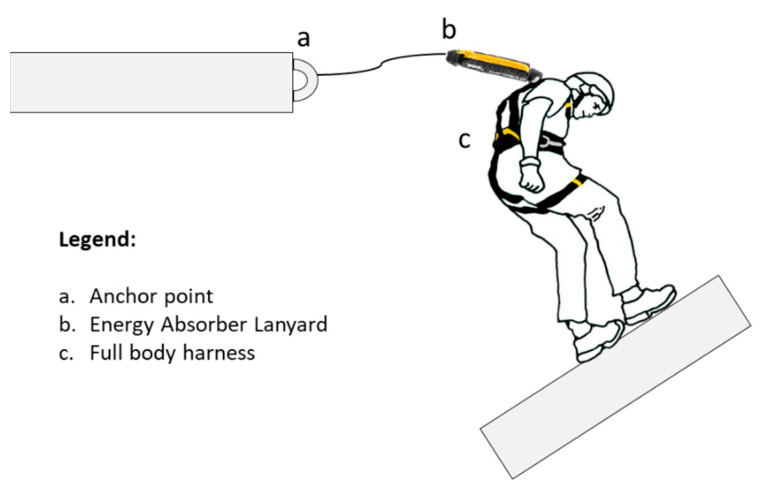
Typical fall arrest systems (FAS).

The FAS is designed according to standard EN 363 [10] to stop a fall safely [11,12,13]. Figure 1 shows a simple FAS, comprising (a) anchor point, (b) energyabsorber lanyard (EAL), and (c) full body harness.

EAL are made up of three parts: (a) the energy absorber (EA), (b) the lanyard (L) [14], a linear element that gives length to the equipment, and (c) connectors, which allow it to be attached to the harness and to the anchor point. The EA and L are inseparable without rendering the equipment unusable and can be supplied either with or without connectors. Most EAs are made of textile tape, of various widths (20–50 mm), usually of high-tenacity polyester or polyamide stitched with polyester yarn in a protective heat-shrinkable polyethylene or woven case, although slight variations can be found in terms of the material of the outer sheath (fabric or steel mesh) and the type of stitching, which can be found across the entire width of the tape or just sewn on one side. The diameters of the linear element, usually formed by synthetic fibers, in the form of a rope with a sheath and core or plaited rope structure, fall in the range 9–16 mm. The sheath is usually made of polyester and the core from polyamide, if rope; or polyester and/or polyamide if they are tape. The rope terminations are sewn to form a loop and are protected against abrasion with plastic parts. The linear element can also be made of tubular polyamide tape and houses elastic tape inside, or more unusually it can be formed with 8 mm steel cable (common in tree-trimming work and others where cutting tools or saws are used). The join between the absorber element and linear element is made with two interlocking bands, the seams are usually protected with a polyethylene cover. The connectors used are made of steel or aluminum alloys, with a self-locking closing mechanism, with a minimum of two different voluntary actions to achieve their opening. The breaking forces of the connectors in a static slow traction test usually exceed 25 kN on the major axis.

The EAL plays the main role in dissipating the kinetic energy generated during a fall from a certain height [15]. It is of vital importance to ensure that arresting forces do not generate harmful values, and it is a moral duty for engineers to ensure that these forces are kept at the lowest possible values. International regulations establish two types of EA; for fall factor 1 (FF 1) and for FF 2. Type 1 ISO 10333-2 [16] and 6 ft free fall distance of the ANSI/ASSP Z359.13 [17] are designed for FF 1. EA Type 2 ISO 10333-2 [16] and EA 12 ft free fall distance of the ANSI/ASSP Z359.13 [17], in line with EA AS/NZS 1981.1 [18] and EA certified according to Standard EN 355 [19], are designed for fall factor 2 (FF2). The latter are those commonly used in Europe, Asia, and Russia and used exclusively for any fall factor, FF1 and FF2.

The requirements of the different standards can be observed in Table 1. In Europe, Standard EN 355 [19] establishes 6 kN as the maximum arrest force (Fm) that the worker must receive in fall arrest (corresponds approximately to a deceleration of 6 g (gravitational acceleration 9.81 N/m^2^) for workers weighing 100 kg). We see that other regulations limit Fm to 8 kN and average deployment force (Fa) to 80% Fm during fall arrest. These maximum values have been discussed in the studies undertaken by Clawford [20] and Sulowski [21,22], where the importance of achieving values close to 4 kN is highlighted in order not to suffer injuries in falls on any axis of the body, mainly in lateral falls. Only Standard CSA Z259.11.17 [23] limits the maximum acceleration (Am) and the average acceleration (Aa) the user can receive.

The minimum necessary energy absorption capacity (E) estimated according to (1) and expressed in kJ is included in Table 1 along with the regulatory requirements related to the test mass (m) in Kg, the free fall distance (h) in meters, and the maximum free fall distance allowed (X) in meters. Table 1 shows that the American standard ANSI/ASSE Z359: 13 [17] is the most stringent in terms of energy absorption, followed by the European standard. CSA Z259.11.17 [23] limits the free fall distance in the test to a value between 0.7 and 0.95 to the maximum free fall distance declared by the manufacturer (Xa).

The minimum energy absorption needed to not achieve the Fm can be estimated according to Equation (1).

E = mgh(1)

where E is potential energy (J), m is user mass (kg), g is acceleration of gravity (9.81 m/s^2^), and h is free fall distance (m).

**Figure 2:**
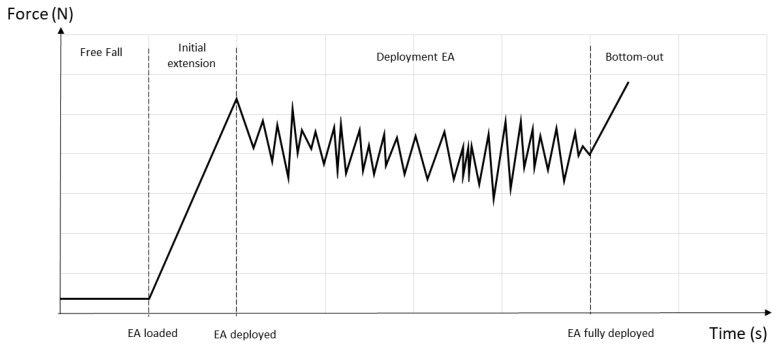
Dynamic performance of an energy absorber (EA) [23].

No studies have been found on EAL performance to act as a base, and no relevant information from manufacturers has been found. Miura and Sulowski, in 1991 [24], presented a graph (Figure 2) on the idealized performance of an EA. Wu [25] studied E4 Z259.16, low-capacity energy absorber. Goh [15] more recently carried out an empirical study on EAs marketed under Standard AS/NZS 1891.1 [18], concluding that Fm basically corresponds to the activation force when EAs are studied as components. For the design of FAS it is essential to know the Fa. Fa is necessary to estimate the elongation that the absorber will suffer during the fall and therefore essential data to calculate the clearance distance. This work provides empirical data required for the design of FAS with EAL in accordance with EN 355 [19].

This paper was developed with the aim of providing experimental data that is useful for the design of EAL and for setting regulatory requirements of EN 355 [19]. It is necessary given the little relevant information provided by manufacturers [15,26], as well as the lack of empirical tests on this equipment that provide Fm and Fa values. The Fa is a necessary parameter to estimate the user’s fall distance and thus achieve a correct design of the EAL [15]. In addition, it is very useful to contrast the values required by the different design standards to provide data to the technical standardization committees so that, taking into account the conclusions, they can include improvements in equipment on the market and establish more demanding and therefore safer requirements for the user. It is essential to reduce the force of impact that the user receives in fall arrest to the lowest value facilitated by technological advances.

## 2. Materials and Methods

The test samples have been selected covering the different types of EAL energy absorbers integrated in lanyards available on the market and certified according to EN 355 [19]. Two of the authors have more than 20 years of experience in FAS, employed in the construction field and in the state fire department. They have extensive knowledge of the market and the available manufacturers that commercialize EAL according to EN 355 [19]. They have been in charge of making the selection of equipment covering various models, types, and manufacturers most commonly used. Table 2 includes specimen data. The selection includes products from six different manufacturers that distribute their products internationally.

A total of 20 EAL were tested; two samples from each of the ten pieces of equipment were studied. The specimens were coded with two numbers separated by a hyphen: the first number represents the equipment type: (1) rope and energy absorber; (2) adjustable rope and energy absorber; (3) strap and energy absorber; (4) elastic strap; (5) elastic strap and energy absorber; (6) wire rope and energy absorber; and the second number represents the specific specimen within the type. The chosen coding allows us to unequivocally identify the specimen; this code is also used to identify the tests. The test was performed twice for each model, each time with new equipment. Thus, sample 2−2 corresponds to an adjustable rope-type EAL, the second specimen of this type. Likewise, 2−2-II corresponds to the second test carried out on the code 2−2 sample.

Personal protective equipment is governed by strict production regulations, as set out in Regulation (EU) 2016/425 [27] which requires quality control that guarantees homogeneity in the manufacture of this equipment, therefore, a small sample was accepted for the number of trials.

In cases 1−1, 1−3, 4−1, and 6−1, in which the manufacturer does not supply the connectors, the instruction manual was followed to choose suitable connectors, ones with EN 362 certification have been used [28] with 38 kN of rupture on the major axis in static strength testing, 10 cm in length and made of steel. Therefore, 20 cm has been added to the length declared by the manufacturer.

This paper analyzes the process of arresting a worker’s fall and specifically the force–time curves obtained in the dynamic tests carried out. The simulated FAS (Figure 1) corresponds to the simplest and most commonly used [29,30]. The study of this FAS is of great interest given the lack of information on Fa and Fm in EAL and the scarce relevant information provided by manufacturers [26].

**Figure 3:**
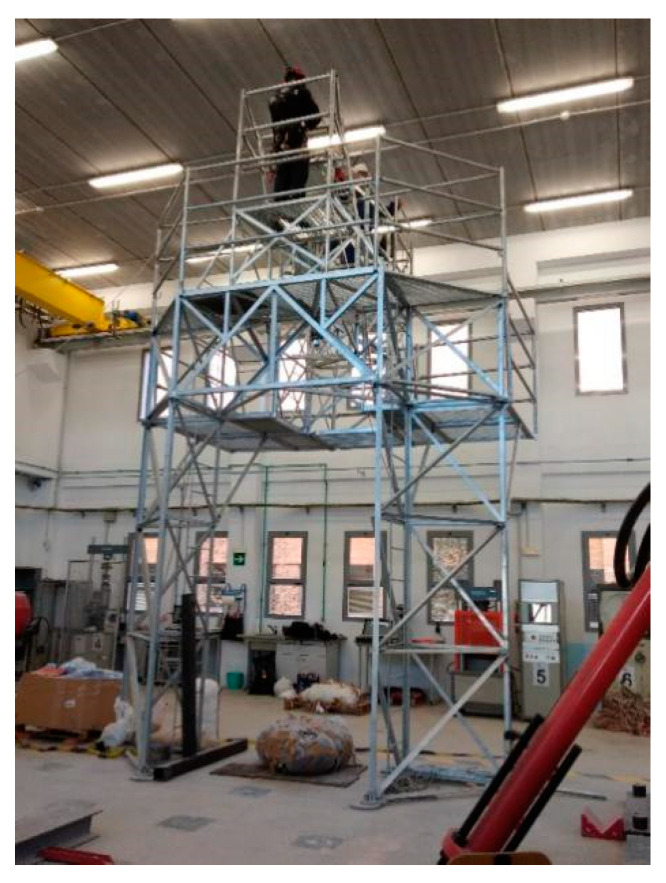
Steel structure for testing.

The experiments were carried out in the Large Structures Laboratory of the Department of Civil Engineering of the University of Alicante. The gantry, designed by the authors, was used for dynamic trials (Figure 3) and has already been successfully tested in previous research [14,31]. The gantry is equipped with a spherical ball and hinge that allows free oscillation during fall arrest, as set out in EN−364 [32] and EN−355 [19] protocols, and complies with the requirements of the regulations for performing accreditation tests. The RSCC-type load cell is made by HBM [33] and it resists a maximum force of 50 kN. The control and data analysis software used was PCD2K from Servosis Testing Machines [34]. This software has a control frequency of up to 40 kHz.

**Figure 4:**
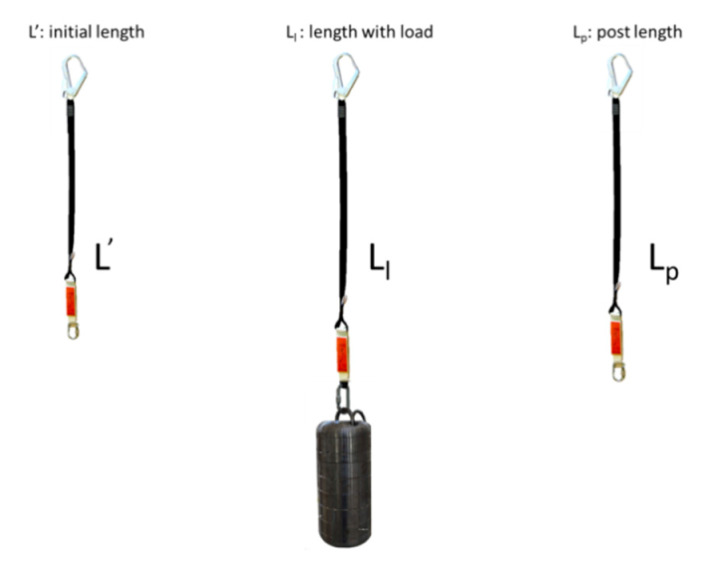
Measured lengths.

Prior to carrying out the tests, all the equipment was subjected to temperature and humidity conditioning for 24 h. Likewise, before carrying out the dynamic tests, compliance with the regulatory requirement of static preloading was verified in accordance with point 5.3.2 of EN 364 [32], for this it is necessary to measure the length of each lanyard before and after a load of 2 kN, that corresponds to suspending a mass of 204 kg. The length of the equipment was obtained by direct measurement with a flexometer on the samples suspended from the gantry, ensuring that the equipment was extended, but without a load, Figure 4. The initial length of the absorber integrated in the anchor equipment was recorded, measuring from the two opposite points that receive the load with connectors (L’).

For static preload, with a mass of 204 kg, as specified by standards EN 355 [19] and EN 364 [32], the lanyard was placed on the test gantry and the mass was suspended for 3 min at the lower end, recording the loaded length (Ll). The load was removed and the length (Lp) was measured again, once the load with the equipment suspended had been removed.

**Figure 5:**
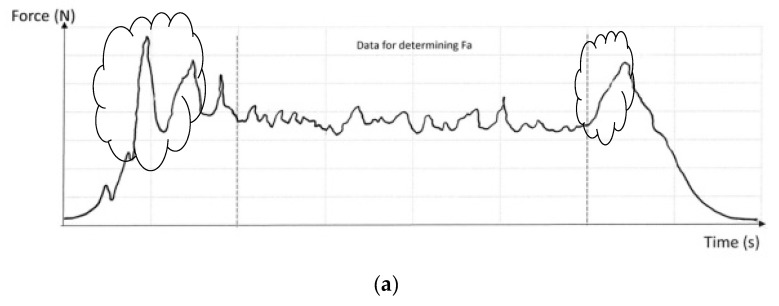

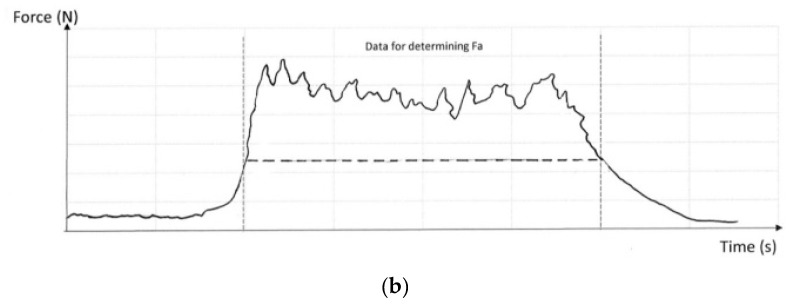
Criteria for calculating Fa: (**a**) Goh [15]; (**b**) ANSI/ASSP Z359.13 [17] (clause 4.1.10).

One of the following two criteria can be followed to determine the Fa: the criterion used by Goh (2014), using data only from the deployment of the absorber, (Figure 5a), not considering the activation force nor the values of the forces that bring collapse or bottom-out; or the criterion set out in clause 4.1.10 of ANSI/ASSP Z359.13 [17] that, unlike Goh, decides to take all the values between the first and the last points that take a value of 2.2 kN (Figure 5b).

In both of the above criteria [15,17], we find that curves for EA (without lanyard) and the length (2 m) necessary for the dynamic test of the EA are achieved by adding a chain (quasi-rigid element without deformation or energy absorption). In this study, we analyzed EAL, and their curves are significantly smoother than those represented by Miura [24], Figure 2, or Goh [15], Figure 5a. In the case that concerns EAL, the peaks do not appear at the beginning and end of the curve (the clouds in Figure 5a). In the calculation of Fa in the EAL in the case of fall factor (FF) 2 with 100 Kg of mass, the criterion used is indistinct. We highlight the importance of 100 Kg of mass, since with this mass the equipment must not reach the end of the absorber path. As a consequence, we have taken all the force values between 2.2 kN as calculated in ANSI/ASSP Z359.13 [17], and only the results of the equipment that meets the regulatory requirements have been taken into account.

**Figure 6:**
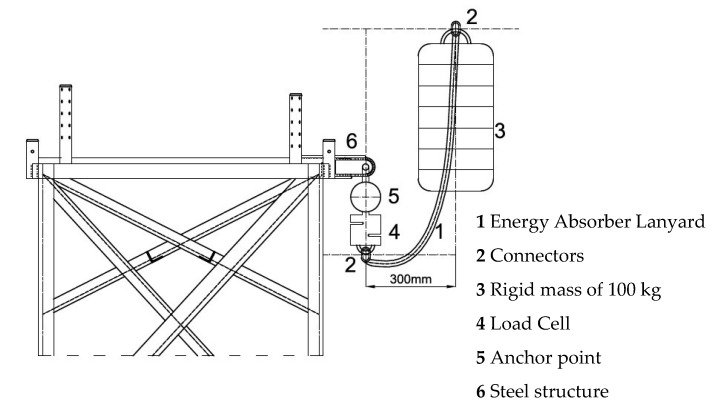
Dynamic behavior testing procedure.

Figure 6 shows a sketch of the test procedure according to EN 355 [19]. For the re-creation of the dynamic tests, the worst possible scenario allowed by the equipment has always been considered, that is, a fall of FF 2. First, the absorber integrated in the anchor equipment of the load cell of the test gantry was suspended, from the lower end we suspended a mass of 100 kg. It was raised as high as the test specimen allows to a maximum horizontal distance of 300 mm and dropped. The adjustable EAL (code 2−1 and 2−2) have been tested to the maximum extension length allowed by the equipment. The software used [34] allows the recording of 10,000 results per test for 10 s. The force–time curve was obtained for each test, and the curve was subsequently analyzed, highlighting Fm, t1, and t2, being t1 the instant in which it begins to acquire tension and t2 the instant in which Fm is reached. The Fa was calculated for each test, it is the average of the forces from reaching 2.2 kN the first time and until that value is reached again.

For each recorded moment (10,000 for each test), the force balance (Equation (2)) was applied, obtaining the instantaneous accelerations (Ai) and therefore the time–acceleration curve, the maximum acceleration (Am) and the average acceleration (Aa) for each test performed.

A_i_ = (F_i_/m) − g (2)

where Ai is acceleration at a given instant (m/s^2^), Fi is force at a given instant (N), m is user mass (kg), and g is acceleration of gravity (9.81 m/s^2^)

Independent variables that affect the process of stopping a fall with FAS are the length of the EAL and the mass free-falling height. The mass in this case is a constant, 100 kg. The dependent variables that we can obtain will be the maximum arrest force (Fm) and deployment average force (Fa). Table 3 lists the independent variables for each experiment.

The theoretical relationship between variables (Equation (3)) is based on energy balance, the work done by the EAL will be equal to the potential energy of the mass.

FaX = mg (h + X)(3)

where X is EAL extension (m), g is acceleration of gravity (m/s^2^), m is the mass (kg), and h is free fall distance (m).

Dependent variables Fm and Fa may be influenced by erratic tearing of the energy absorber stitch, even by some deformation in metal elements of FAS (connectors and buckles) that has been disregarded in the present work. This method has previously been successfully employed by the authors [14,31] and also employed by other authors in their work considering, for example, shock absorbers, harnesses, and even a multicomponent system [12,21,35,36,37].

## 3. Results

The results obtained, expressed in this section, are studied in comparison with the current regulatory requirements in Europe [19]. A typification of the force–time curve is presented in the phenomenon of fall arrest with an EAL of FF 2 and 100 kg of mass. The results obtained and their influence on the impact that the user would receive are analyzed. Firstly, and only for testing purposes, we subjected the equipment to 24 h of acclimatization and subsequently to the elongation test established by EN 355 [19].

### 3.1. EAL Static Preloading

Satisfactory results were obtained, see Table 4**,** when carrying out the test described in Section 5.1 of EN 355 [19], the permanent elongation, due to the activation of the EAL after a preload of 2 kN, did not exceed 50 mm in any case. The data in Table 4 are the average results of the two samples of each model. Where L’ is EAL initial length with connectors and Lp is EAL length after test with connectors.

The extension range obtained is between 5 to 30 mm, obtaining an average of 16.81 mm. It is also observed in the table that the dispersion cannot be attributed to a certain type of EAL. The same type (rope + EA) has a permanent extension range under 2 kN of load between 5 and 30 mm. The nonactivation of the EAL under 2 kN load serves to determine that during the use of the EAL under minimum load, such as the weight of a person or an involuntary pull of the system, the absorber will not start to deploy or activate, thus keeping all its energy absorption capacity intact in the event of a fall.

### 3.2. EAL Length

For equipment not sold with a connector (codes 1−1, 1−3, 4−1, and 6−1), a standard EN 362 [28] steel connector has been provided. In the case of EAL with adjustable ropes (codes 2−1 and 2−2), the measurement has been taken at its maximum length.

The lengths of the absorbers integrated in the lanyard (Table 5) vary in each selected sample, we have samples in a range between 91 and 207 cm in initial length with connector, the different lengths allow a free fall distance between 182 and 414 cm (double length).

Initial length measurements reveal that (with standard 10 cm connectors) in cases 1−2, 2−2, and 6−1, the total length with connectors exceeds 2 m allowed by most standards (except CSA Z259.11.17 [23] that does not include this requirement). Subsequently, in Section 4.2, the influence of this data with respect to the fall arrest Fm will be studied.

Samples 1−1 and 5−1 highlight the large difference in length of the equipment indicated by the manufacturer in its manual with that actually measured in the laboratory. In both cases, the actual lengths of the equipment measured in the laboratory are substantially less than the lengths declared by the manufacturers (11.5% and 28.5%, respectively). Three samples (1−2, 2−2, and 6−1) exceed the 2 m required by most standards, and samples 1−4, 2−1, and 4−1 would be in accordance with the standard and with a variation of less than 2.5% with respect to that declared by the manufacturer.

### 3.3. Energy Absortion, Fm y Fa

The maximum drop height allowed by each sample (h) and the minimum absorption capacity are presented in Table 6 for each of the PPE studied. The maximum height of fall has been calculated by the authors as double the measured length (Li * 2), on the EAL with connectors in extension, but without load. The minimum energy absorption capacity that each equipment must have has been estimated according to (1) and is in the range of 1.78 kJ and 4.06 kJ.

Four of the samples (1−2, 2−1, 2−2, and 6−1) exceed the energy established by EN 355 [19] for absorbers as components. This is so because they exceed the maximum length allowed for this type of equipment.

**Figure 7:**
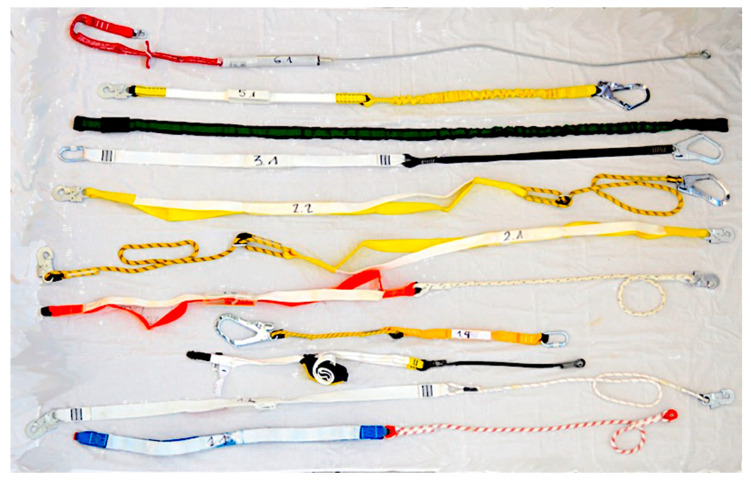
Samples after dynamic performance test.

Three of the ten samples (1−2, 2−2, and 6−1) require the absorber to dissipate more energy than the minimum required by the 3.92 kJ standard, this is because the actual length of the equipment is greater than the two meters maximum contemplated by the standard. The h in expression (1) is greater, and the energy to be absorbed will also be greater. However, in these cases (1−2, 2−2, and 6−1), the manufacturer has managed to absorb the extra energy generated (Table 6) and keep the Fm within acceptable values. The absorbers did not rupture completely, as shown in Figure 7. They would have been able to absorb more energy by either increasing the drop height or the operator’s mass. The requirement not to exceed 2 m in length is unfounded from the FM point of view in the arrest process; although it is important when establishing the clearance distance (distance necessary to avoid hitting the ground). In this regard, CSA Z259.11.17 [23] has removed the maximum length requirement but maintains limitations on Fm and Fa.

**Figure 8:**
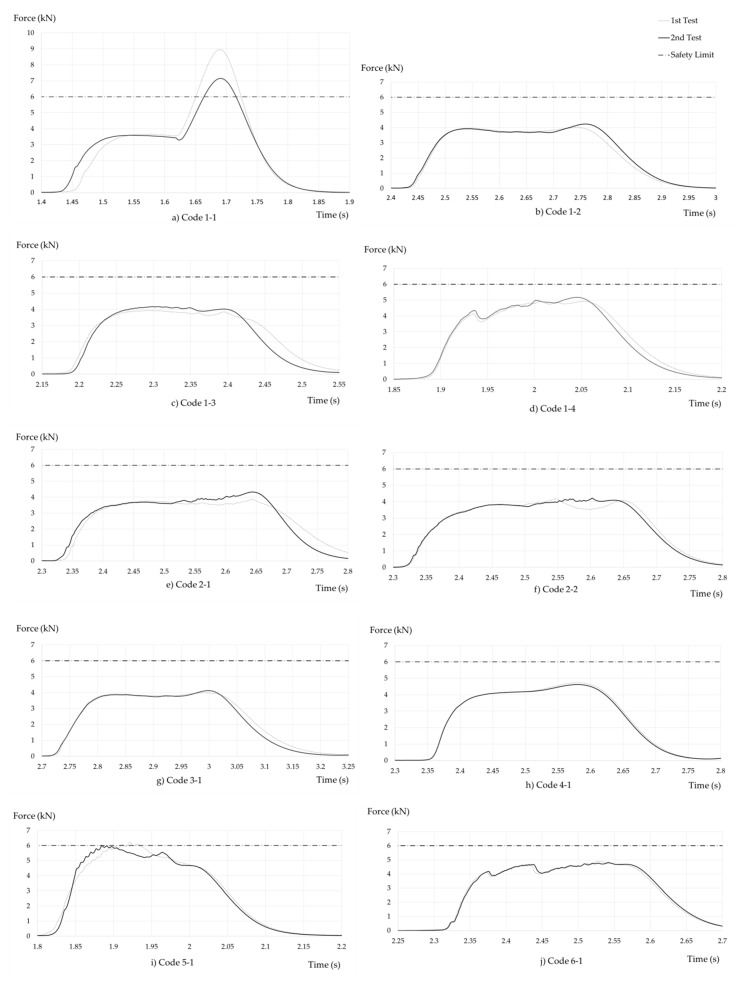
Time–force charts.

Figure 8 shows the curves obtained in the dynamic performance tests. Each graph contains the curves of the two tests carried out on the same model. Both curves have been temporarily matched, and the regulatory limit for better data compression has been represented. It can be clearly seen that the code 1−1 sample exceeds the Fm of 6 kN value in both tests carried out (1.1.I and 1.1.II). The dispersion in the results of both samples is especially significant with respect to the rest of the results obtained, where the curves are practically identical in both tests. Sample 1−1 reached bottom-out without absorbing the energy generated during the fall. The stitching is appropriate; as shown in the graph, the equipment begins to sew at approximately 3000 N, but it lacks distance, and once the seam is finished the absorbent tape snaps. It can be seen in the graph that once the unstitched tape tears, the “safety tape” starts to act and a force peak of values incompatible with its use is generated. Sample 5−1 acts at greater forces than those expected, the bad sewing calibration causes it to begin to tear (start of energy absorption) near 6000 N. The results obtained from these two samples have not been taken into account when calculating the mean statistical data of Fm and Fa. They are not considered since they should not be on the market because they do not meet the requirements of the EN 355 standard [18] and therefore do not comply with the EU Regulation [27]. Considering them would distort the data of the study carried out.

**Figure 9:**
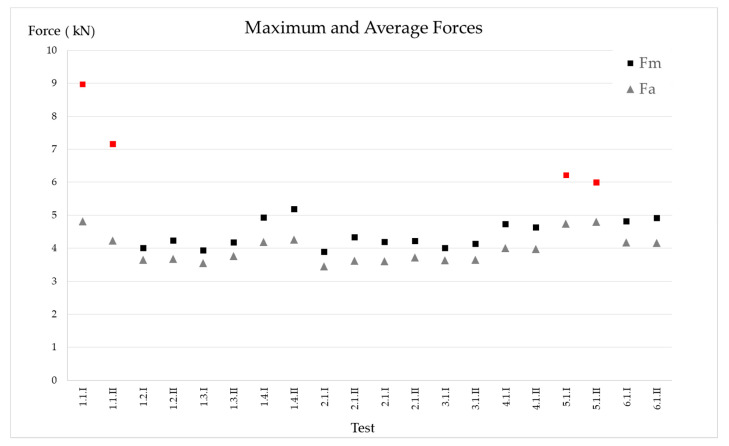
Average and maximum arrest forces.

Figure 9 shows the maximum and average forces obtained for each test. It can be seen that (except for 1−1 and 5−1, marked with red squares) it is practically possible to fall below 5 kN of maximum force, and also the absorber was not completely deployed.

Table 7 shows the statistical data for Fm, Fa, the time to reach Fm, and the rate of application of the load (including only those that comply with EN 355 [19], that is, the values of Fm obtained in the tests on code 1−1 and 5−1 have not been considered). As can be seen, it is feasible to limit the Fm below 5 kN and the Fa to 4.3 kN. The load application speed exceeds 38 kN per second. The loading speeds have already been approximated by Carrión [29] and Irles [37] by numerical methods, at the expense of experimental data for the calibration of some parameters such as damping. The experimental results obtained here will allow this calibration.

We have four results that do not comply with EN 355 requirements, those that we obtained from tests with code 1.1 and code 5.1. On the other hand, we have 16 results that conform with EN 355.

We first compare the 4 values with the 16, to see if we can say that they have the same distribution. To do this, since the size of the two samples is very small, 4 and 16, respectively, we propose to make a nonparametric contrast of equal distributions. We have applied the Kruskal–Wallis test [38] and the concordance test [39], obtaining the following results (Table 8).

Both tests conclude that there is significant evidence that the two sets do not come from the same distribution; we can reject the equal in the Fa distribution for the two sets

Below we analyze the set of 16 data to see if the regulatory requirements are adapted to the process of stopping the fall with an EAL or if they are too conservative and may require a higher level of security.

**Figure 10:**
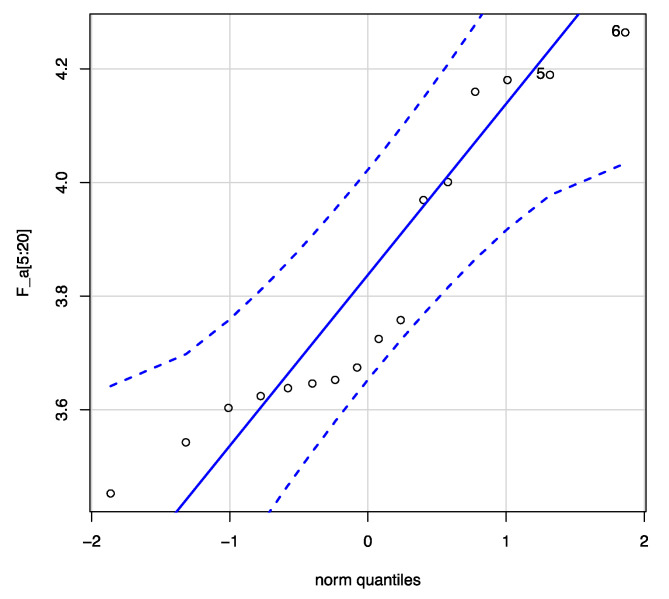
Normal Q–Q plot for Fa.

The descriptive statistics for Fa (16 samples) are minimum, 3.453; first quartile, 3634; median, 3.700; mean, 3.818; third quartile, 4.041, and maximum, 4.264 N. In order to analyze this sample, it is interesting to know if the distribution of the values of the mean forces obtained follow a normal distribution. Figure 10 compares the probability distributions of our sample with the normal distribution. Now, let us see if we can assume that the sample comes from a normal population.

Shapiro–Wilk normality test gives us a test to contrast whether or not a sample comes from a normal distribution. The test result applied to the 16 values provides the following for Fa: W= 0.88065, *p*-value = 0.03972. We must reject normality (Figure 10) in the data as we get a very small *p*-value, less than 5%. If we do not accept the normality hypothesis, we must apply a nonparametric test to validate the hypothesis that the Fa is, for example, 4.8, 4.35, or 3.9 kN. Table 9 shows the results obtained by applying the Wilcoxon [40] test to our data.

Wilcoxon test provides evidence that the estimate Fa = 80% Fm is not sustainable; and it can be said to be below 4.35 kN. The main difference between code 1.1 and other devices is that code 1.1 had too short an energy absorber length. When the tearing finished (Figure 8a), the forces reached unsafe values, over 6 kN.

Figure 8i shows an EAL whose sewing turns out to be badly calibrated, the force necessary for it to start tearing is very close to 6 kN. Whether an EAL is able to keep the Fm below 6 kN and therefore to be safe depends on the characteristics of the energy absorber (type of stitching and absorber length).

A Fm greater than 6 kN on the worker could result in serious injury, even death [19,22].

### 3.4. Tipification of the Force–Time Curve in the EAL

**Figure 11:**
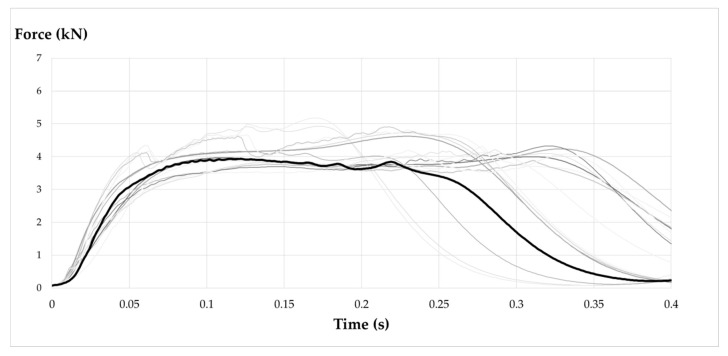
EAL time–force curve.

The performance of the EAL obtained in the different tests has been outlined in Figure 10. The main difference observed with the EA studied by Miura, Sulowski, and Goh is that the peak corresponding to the activation force at the beginning of the deployment in the EAL (Figure 5a) does not appear. By introducing a deformable element such as the lanyard (EAL) in the EA, it is possible to reduce the force peak, flattening the force–time curve (Figure 11), making it smoother and therefore less harmful to the user.

The curves obtained in the tests carried out have been left in gray, and the typical force–time curve in arresting a factor 2 fall of 100 kg with an EAL has been highlighted. A typical curve is obtained as the average of experimental force–time charts. It can be seen that the arrest phenomenon lasts for approximately 0.35 s and presents a plateau of Fa value below 5 kN, from there, successive rebounds of much smaller magnitude occur until the total arrest of the ballast.

### 3.5. Accelerations

Applying equation (2), the accelerations have been obtained for each instant (Ai) in which force data has been recorded (10,000 per test). As we are in a case of free fall, a practically vertical acceleration is assumed. We obtained the time–acceleration curves for each piece of equipment tested. The statistical data for the maximum acceleration (Am) and the average acceleration (Aa) are shown in Table 10, without including the 1.1 and 5.1 pieces of equipment that, as previously mentioned, should not be on the market for not complying with the requirements and are therefore potentially dangerous.

The maximum accelerations obtained are in the opposite direction to gravity. These decelerations are limited only in the Canadian CSA Z259.11.17 standard [23]. All samples tested meet the requirements stipulated; Am ≤ 10 g and Aa ≤ 7 g. It was expected that the requirement would be fulfilled since the equipment tested is certified under the European standard, which is much more restrictive than the Canadian equivalent in terms of the Fm allowed.

Table 11 shows the results for each test, the most prejudicial being 4.3 g (except 1.1 and 5.1) and the least prejudicial being 3 g, which are acceptable values in both cases.

**Figure 12:**
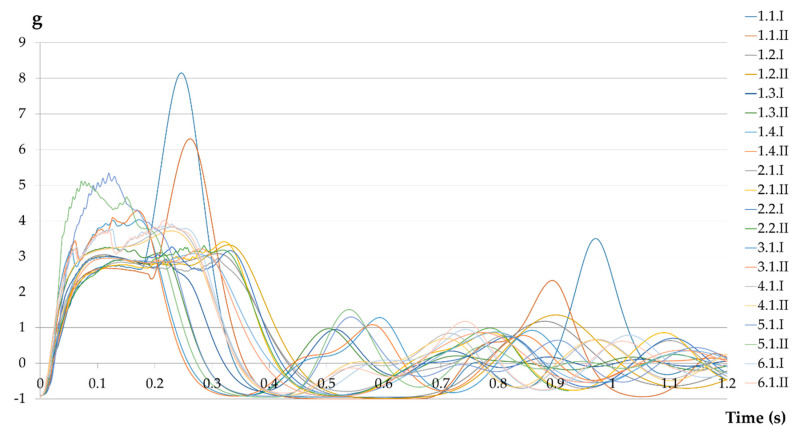
Mass acceleration (g).

Regarding the application times of these accelerations, in no case is the deceleration more than 0.1 s above 8 g. Only the sample code 1.1 reaches the value of 8 g, which, as already mentioned, should be withdrawn from the market together with 5.1. In the rest of the tests, the ballast has suffered decelerations between 3 and 4 g for a period between 0.3 and 0.4 s. Figure 12 shows the acceleration undergone by the ballast as a function of time for each test.

We have two sets of results, the first comprises four results from devices not complying with EN 355 requirements, those that we obtained from tests with code 1.1 and code 5.1, and the second comprises 16 results from devices that conform with EN 355. We compare (Table 12) the 4 values with the 16, to see if we can say that they have the same distribution.

Both tests conclude that we can reject the equal in the Aa distribution for the two sets

We go on to analyze the set of 16 values, and we will check how rigorous is the regulatory requirement that allows Aa 7g. The descriptive statistics for Aa (16 samples) are as follows: minimum, 24.73; first quartile, 26.54; median, 27.20; mean, 28.43; third quartile, 30.84, and maximum, 32.84 m/s2.

**Figure 13:**
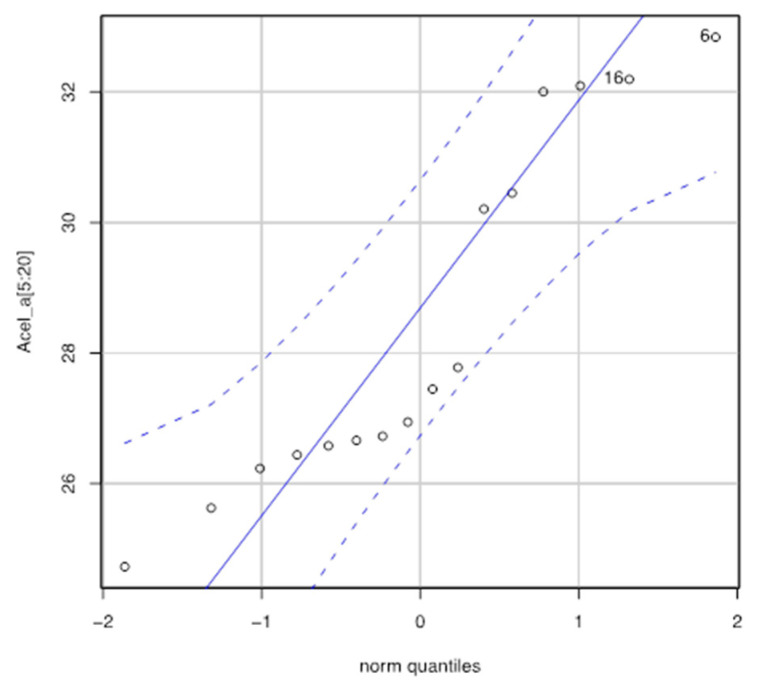
Normal Q–Q plot for Aa.

Let us see if we can assume that the sample comes from a normal population. Shapiro–Wilk normality test = 0.87082, *p*-value = 0.02799. We must reject normality (Figure 13) in the data as we get a very small *p*-value, less than 5%.

If we do not accept the normality hypothesis, we must apply a nonparametric test to validate the hypothesis that the Fa is, for example, 68.6, 40, 30 or 29 m/s^2^. Table 13 shows the results obtained by applying the Wilcoxon [40] test to our data:

We can reject Aa equal 7 g (68.6 m/s^2^) during the process of stopping the fall with EAL, and we can also reject 3.06 m/s^2^.

## 4. Discussion

Fa obtained on EAL with EN certificate ranged from 3.5 to 4.3 kN, higher than described by Wu (2011) and by the North American Standards but similar to Australian EA (Goh 2014). Australia and Europe use high-capacity absorbers, those valid for fall factor 2.

Z359.6 and Z259.16 states that Fa can be estimated at 80% of Fm (6 kN). This would be 4.8 kN, but the data obtained makes us reject that estimate. For European EALs, the Fa would remain at 3.8 kN on average, similar to the 3.9 kN calculated by Goh for Australian EA.

CSA Z259.11.17 requires maximum acceleration to be below 10 g and average acceleration cannot exceed 7g, the findings of this study show that these limits are very conservative. The maximum acceleration obtained is 3.35 g with the average of 2.77 g, far from regulatory requirements.

The analysis of the requirements of the different standards shows us that the pieces of equipment manufactured under EN 355 [19] are safer than those manufactured under ANSI/ASSP Z359.13 [17] and CSA Z259.11 [23]. In Europe, Australia, New Zealand, and the International Organization for Standardization [16], the Fm is established at 6 kN. In contrast, in America and Canada, the maximum permitted force is 8 kN. Sulowski [22] demonstrated that these 8 kN could be especially damaging if the fall was not on the axis of the spine (this cannot be guaranteed in an accident), also if the retention was lateral, Sulowski determined that above 4 kN a significant injury was likely to appear.

The research carried out shows that the standards are conservative and may be more demanding and avant-garde in terms of the maximum arrest force allowed. The data obtained allow reducing the maximum permitted force to 5 kN.

EAL certificated EN 355 [19] deploy at an average force of 3.5 to 4.3 kN. Engineers must take the lower value of 3.5 kN to calculate deployment length X (equation 4), so clearance distance is longer and more conservative.

X = mgh/(Fa−mg)(4)

As was expected, there is not a big difference between the American standards and that of Wu [25], because Wu is focused on EA with low capacity. There are not big differences between EN 355 [19] EAL and those EA certificated in Australia and New Zealand (Table 14). But there is significant difference between this two groups. It would be interesting to research deployment average force for EAL certificates under Canadian Z259.16 [41] and American Standard Z359.6 [42].

It should be noted that two of the ten selected models should not be on the market under European certification because the Fm obtained in the tests has exceeded 6 kN. This means that 20% of the analyzed equipment does not meet regulatory requirements. This can cause serious injury to workers and therefore cannot be considered safe PPE. It denotes the need to impose more quality controls. It would be advisable to increase the number of samples selected to check if the value of 20% of noncompliant equipment is maintained.

It should be noted that this studio is limited to EAL certified to EN 355. Findings cannot be useful for other EA or EAL. It can be assumed that the vast majority of manufacturers produce homogenously. (Figure 8). It can also be assumed that the free fall distance does not significantly affect the Fa.

It would also be interesting to extend this study to EAL certified under different regulations to European ones. This work presents the typification of the force–time curve for EAL with a fall of 100 kg in a fall factor 2. Expanding the study to different masses to contemplate the greater range of workers would provide a greater degree of knowledge and elimination of possible injuries in people of low or high weight.

The time-based Fa has been calculated, but the displacement-based calculation would be interesting. This would be achieved by filming the fall arrest process with high-speed cameras. You could determine the elastic deformation of the equipment and how this influences the safety distance, since all the standards that establish requirements do so on the plastic deformation, neglecting the elastic.

## 5. Conclusions

All the tested equipment has successfully passed the absorber activation test, none of them showed permanent deformations greater than 50 mm.

The length of the EAL has presented variations by excess (up to 7 mm) or by default (up to 57 mm) of the length declared by the manufacturer. This fact is not relevant in terms of user integrity. Exceeding the 2 m set by the regulations does not affect the maximum braking forces. It seems interesting to eliminate the requirement of EN 355 [19] as CSA Z259.11.17 [23] does, where it is the manufacturer that decides for what mass and what length its EAL keeps the forces and accelerations below the established values.

Increasing its length and need to absorb more energy (by increasing the height of free fall) has not been a problem either, since most pieces of equipment tested ended up with part of the absorber being undeployed.

The limit of 6 kN for Fm in falls of 4 m with 100 kg of mass is perfectly achievable with current technology. The results obtained indicate that this limit for the EAL can be reduced by 16.6%, establishing that the Fm is equal to or less than 5 kN. This value is especially significant when analyzing the requirement of the American and Canadian standards. In the latter case, the Fm would go from 8 kN to being limited to 5 kN, representing a 37.5% reduction in the impact force that a user would be allowed to receive in fall arrest with an EAL.

Fa turns out to be a requirement that is not required in EN 355 [19]. It is significant data for the design of the EAL. The Fa turns out to be 87.5% of the Fm for the pieces of equipment studied in this paper, all of them according to EN 355 [19]. The estimate based on 80% of Fm (according to CSA Z259.16 [41] clause 7.3.3.2 or ANSI/ASSP Z359.6 [42] clause 6.3.3.2) should not be used for EAL certified according to EN 355 [19].

The actual application speeds of the load in the free fall have been obtained, reaching over 38 kN per second. This information is relevant to the design of each component involved in an FAS. These must be designed to withstand these loading speeds. The data obtained are necessary when conceptualizing standard testing.

The typification of the force–time curve for an EAL in a fall of FF 2 and 100 kg of mass is presented. This curve represents how these units perform. The quasi-constant plateau that involves the ripping of the absorber can be clearly seen. A relevant conclusion is that in all the studied cases, the two tests on the same model yielded virtually identical results. Therefore, homogeneity in the production of manufacturers of this personal protective equipment is highlighted

After studying the data obtained, the following adjustment is proposed in the requirements established by EN 355 [19] for EAL. The Fm can be reduced to 5 kN, representing a reduction in impact of 16.6%. The Fa can be estimated as 87.5% of the Fm. (Fm 5 kN and Fa 4.3 kN). It is proposed to eliminate the maximum equipment length requirement and to establish the requirements limiting the force the user receives in fall arrest. The review committee of EN 355 [19] would have no problem in being able to limit the maximum deceleration that the ballast must undergo during the fall arrest to 4.1 g. Limiting the maximum deceleration to 4.5 g turns out to be a reasonable value after studying the results obtained.

This study provides sufficient experimental data for the calibration of numerical models based on finite elements. The numerical calculation for the design of components with numerical methods represents an advance compared with the experimental tests, given the lower cost and higher speed of obtaining results once a proven and reliable numerical model has been obtained.

Lastly, the results obtained indicate the need for an increase in the production control of this equipment, basically because 20% of the equipment tested does not satisfy the certification requirements of EN 355 [19].

## Figures and Tables

**Table 1:** Requirements of the different standards.

Standard	m (kg)	h (m)	X (m)	E (kJ)	Fm (kN)	Fa (kN)	Am g	Aa g
ISO 10333-2:2000 TYPE1	100	1.8	1.2	1.77	4			
ANSI/ASSE Z359.13−2013	128 ^1^	1.83	1.2	2.30	8	4		
ANSI/ASSE Z359.13−2013	128 ^1^	3.66	1.5	4.59	8	6		
ISO 10333-2:2000 TYPE 2	100	4	1.75	3.92	6			
AS/NZS 1891.1:2007	100	4	1.75	3.92	6			
CSA Z259.11.17 22	MF ^2^	MF ^2^	0.7–0.95 (Xa ^2^)		8		10 g ^3^	7g
EN 355:2002	100	4	1.75	3.92	6			

^1^ Conversion factor 1.1 is being used for comparing rigid test weight to the human body (140 kg). ^2^ Test mass, free fall, and maximum extension (Xa) are the ones that the manufacturer declares in its manual. ^3^ Allowed in the deceleration range of 8–10 g for a cumulative period of no longer than 0.1 s.

**Table 2:** Technical specifications of samples according to the manufacturer’s instruction manual.

Code	EAL Type	Manufacturer	Connectors	Length Declared by the Manufacturer l (m)	L = l + 0.2 (m)
1−1	Rope + EA	A	No	1.8	2
1−2	Rope + EA	B	Yes	2	2
1−3	Rope + EA	C	No	0.8	1
1−4	Rope + EA	D	Yes	0.9	1.1
2−1	Adjustable rope + EA	D	Yes	2/1.5	2
2−2	Adjustable rope + EA	D	Yes	2/1.5	2
3−1	Webbing + EA	B	Yes	1.5	1.5
4−1	Elastic webbing	F	No	1.5	1.7
5−1	Elastic webbing + EA	D	Yes	2	2
6−1	Wire + EA	D	No	1.8	2

**Table 3:** Independent variables for each experiment.

Code	Experiment	EAL Length (m)	Free Fall (m)	Mass (kg)
1.1	Test 1	1770	3540	100
	Test 2	1770	3540	100
1.2	Test 1	2030	4060	100
	Test 2	2030	4060	100
1.3	Test 1	989	1978	100
	Test 2	989	1978	100
1.4	Test 1	880	1760	100
	Test 2	880	1760	100
2.1	Test 1	1960	3920	100
	Test 2	1960	3920	100
2.2	Test 1	2010	4020	100
	Test 2	2010	4020	100
3.1	Test 1	1520	3040	100
	Test 2	1520	3040	100
4.1	Test 1	1660	3320	100
	Test 2	1660	3320	100
5.1	Test 1	1430	2860	100
	Test 2	1430	2860	100
6.1	Test 1	2070	4140	100
	Test 2	2070	4140	100

**Table 4:** EAL preload.

Code	Lp − L’ (mm) < 50 mm	Code	Lp − L’ (mm) < 50 mm
1−1	20	2−2	10
1−2	25	3−1	30
1−3	10	4−1	10
1−4	30	5−1	10
2−1	10	6−1	22

**Table 5:** EAL length.

Code	Li, Measured in Laboratory (mm)	L _initial,_ Declared by Manufacturer (mm)	Difference (mm)	%
1−1	1770	2000	230	−11.5
1−2	2030	2000	−30	1.5
1−3	989	1000	10	−1.0
1−4	880	900	20	−2.2
2−1	1960	2000	40	−2.0
2−2	2010	2000	−10	0.5
3−1	1520	1500	−20	1.3
4−1	1660	1700	40	−2.4
5−1	1430	2000	570	−28.5
6−1	2070	2000	−70	3.5

**Table 6:** Energy absorption and Fm.

Code	H (cm) L’−×2	Minimum Absorption Capacity (kJ)	Absorption Energy (kJ)	Fm (N)	
				Test 1	Test 2
1−1	3540	3.47	No	8969	7155
1−2	4060	3.98	Si	3969	4237
1−3	1978	1.94	Si	3935	4169
1−4	1760	1.72	Si	4915	5177
2−1	3920	3.84	Si	3885	4326
2−2	4020	3.94	Si	4184	4163
3−1	3040	2.98	Si	3999	4127
4−1	3320	3.25	Si	4729	4622
5−1	2860	2.80	No	6214	5983
6−1	4140	4.06	Si	4759	4766

**Table 7:** Force statistics.

Statistics	Fm (N)	Fa (N)	T(s)	V (N/s)
Max	5177	4264	0.333	38,283
Min	3885	3453	0.113	12,364
Mean	4391	3818	0.239	20,228
Median	4229	3700	0.247	18,099
Standard deviation	410	265		
Mean deviation	354	232		

**Table 8:** Kruskal–Wallis and concordance test for Fa.

	Statistic	*p*-Value
Concordance Coefficient	0.969	<0.001
Kruskal–Wallis	8.580	<0.001

**Table 9:** Results of Wilcoxon test for Fa.

Hypothesized Mean t	Alternative Hypothesis	Statistic	*p*-Value	Value Null Hypothesized at 5% sig	Value Null Hypothesized at 1% sig
4.8	<4.8	0	<0.0001	Reject	Reject
4.35	<4.35	0	<0.0001	Reject	Reject
3.9	<3.9	49	0.1742	Accept	Accept

**Table 10:** Acceleration statistics.

Statistics	Am (m/s^2^)	Aa (m/s^2^)
Max	41.97	32.84
Min	29.05	24.73
Mean	34.85	28.88
Median	33.02	27.61
Standard deviation	4.42	2.87
Mean deviation	3.97	2.63

**Table 11:** Maximum and Average Acceleration.

Test	Am	Aa	Test	Am	Aa
1.1.I	8.2g	3.9g	2.2.I	3.3 g	2.7 g
1.1.II	6.3g	3.3g	2.2.II	3.3 g	2.8 g
1.2.I	3.1g	2.7g	3.1.I	3.1 g	2.7 g
1.2.II	3.3g	2.7g	3.1.II	3.2 g	2.7 g
1.3.I	3.0g	2.6g	4.1.I	3.8 g	3.1 g
1.3.II	3.3g	2.8g	4.1.II	3.7 g	3.1 g
1.4.I	4.0g	3.3g	5.1.I	5.3 g	3.8 g
1.4.II	4.3g	3.4g	5.1.II	5.1 g	3.2 g
2.1.I	3.0g	2.5g	6.1.I	3.9 g	3.3 g
2.1.II	3.4g	2.7g	6.1.II	4.0 g	3.3 g

**Table 12:** Kruskal–Wallis and concordance test for Aa.

	Statistic	*p*-Value
Concordance Coefficient	0.844	0.0077
Kruskal–Wallis	6.509	0.0077

**Table 13:** Results of Wilcoxon test for Aa.

Hypothesized Mean t	Alternative Hypothesis	Statistic	*p*-Value	Value Null Hypothesized at 5% sig	Value Null Hypothesized at 1% sig
68.6	<68.6	0	<0.0001	Reject	Reject
40	<40	0	<0.0001	Reject	Reject
30	<30	23	0.0091	Reject	Reject
29	<29	55	0.2641	Accept	Accept

**Table 14:** Deployment average force.

Author	Standard	Min (kN)	Max (kN)	Capacity Absorber
Appendix A	Z359.6	2.67	3.56	FF1 low
Annex A	Z259.16	2.8	3.6	FF1 low
Wu (2011)	E4 EA Z259.16	2.8	3.7	FF1 low
Goh (2014)	EA AS/NZS1891.1	3.2	4.7	FF2 high
Carrión (2020)	EAL EN 355	3.5	4.3	FF2 high

## References

[1] Huang X., Hinze J. (2003). Analysis of construction worker fall accidents. J. Constr. Eng. Manag..

[2] Instituto Nacional de Estadística, Ministerio de Asuntos Económicos y Transformación Digital Accidentes de Trabajo con Baja en Jornada de Trabajo (ATJT), Según Sector y Actividad Económica 2013–2018. https://www.insst.es/accidentes-de-trabajo-y-otros-danos-a-la-salud/-/asset_publisher/JnsaDbNfEi9h/content/accidentes-de-trabajo-con-baja-en-jornada-de-trabajo-atjt-segun-sector-y-actividad-economica.

[3] European Statistical System Directorate-General of the European Commission. Accidents at Work by Sex, Age, Severity, NACE Rev. 2 Activity and Workstation [hsw_ph3_01] 2014–2018. https://appsso.eurostat.ec.europa.eu/nui/show.do?dataset=hsw_ph3_01&lang=en.

[4] Kang M.Y., Siddiqui S., Suk S.J., Chi M.S., Kim C. (2017). Trends of fall accidents in the U.S. construction industry. J. Constr. Eng. Manag..

[5] Wong T.K.M., Man S.S., Chan A.H.S. (2020). Critical factors for the use or non-use of personal protective equipment amongst construction workers. Saf. Sci..

[6] Safe Work Australia (2018). Work-Related Fatalities. https://www.safeworkaustralia.gov.au/system/files/documents/2002/work_related_traumatic_injury_fatalities_report_2018.pdf.

[7] Workplace Safety and Health Institute Workplace Safety and Health Report 2019. https://www.mom.gov.sg/workplace-safety-and-health/wsh-reports-and-statistics.

[8] Ministerio de Empleo y Seguridad Social (2017). Subsecretaría de Empleo y Seguridad Social. Dirección General de Estadística y Análisis Socio-Laboral. Subdireccin General de Estadística. http://www.mitramiss.gob.es/es/estadisticas/monograficas_anuales/EAT/2018/index.htm.

[9] Dong X.S., Largay J.A., Choi S.D., Wang X., Cain C.T., Romano N. (2017). Fatal falls and PFAS use in the construction industry: Findings from the NIOSH FACE reports. Accid. Anal. Prev..

[10] Asociación Española de Normalización (2009). UNE-EN−363:2018, Personal Fall Protection Equipment. Personal Fall Protection Systems.

[11] Arteau J., Giguère D. (1992). Efficiency, reliability and comfort as evaluation criteria for personal protective equipment. Colloque International du Comité Recherche de l’AISS.

[12] Lan A., Arteau J., Sirad C. (2004). Method for validating a multi-component safety system. Saf. Sci..

[13] Carrión E.Á., Saez P.I., Mora-García R.T. (2014). Sistemas de Protección Individual Contra Caídas: Legislación, Definiciones y Equipos.

[14] Pomares J.C., Carrión E.A., Irles R., Gonzalez A., Segovia E.G., Syngellakis S., Schleyer G. (2018). Experimental tests on personal safety devices for height fall. Proceedings of the 15th International Conference on Structures Under Shock and Impact.

[15] Goh Y.M. (2015). Empirical Investigation of the average deployment force of personal fall-arrest energy absorbers. J. Constr. Eng. Manag..

[16] International Organization for Standardization (2000). ISO 10333-2 Personal Fall-Arrest Systems—Part 2: Lanyards and Energy Absorbers.

[17] The American Society of Safety Engineers (2013). ANSI/ASSP Z359.13 Personal Energy Absorbers and Energy Absorbing Lanyards.

[18] Australian/New Zealand Standard (2007). AS/NZS 1891.1:2007 Industrial Fall-Arrest Systems and Devices Part 1: Harnesses and Ancillary Equipment.

[19] Asociación Española de Normalización (2002). UNE-EN−355: 2002, Personal Protective Equipment against Falls from a Height. Energy Absorbers.

[20] Crawford H. (2003). Survivable Impact Forces on Human Body Constrained by Full Body Harness.

[21] Sulowski A.C., Brinkley J.W. (1990). Measurement of maximum arrest force in performance test of fall protection equipment. J. Test. Eval..

[22] Sulowski A.C. (2006). How Good Is the 8kN Maximum Arrest Force limit in Industrial Fall Arrest Systems?.

[23] Standars Council of Canada, Conseil Canadien des norms (2015). CSA Z259.11−17 Fall Arresting Devices-Energy Absorbers and Lanyards.

[24] Miura M., Sulowski A.C. (1991). Introduction to horizontal lifelines. Fundamentals of Fall Protection.

[25] Wu J.Z., Powers J.R., Harris J.R., Pan C.S. (2011). Estimation of the kinetic energy dissipation in fall-arrest system and manikin during fall impact. Ergonomics.

[26] Small G. (2011). Calculating Clearance. www.ishn.com/articles/91975-calculating-clearance.

[27] Official Journal of the European Union (2016). Regulation (EU) 2016/425 of The European Parliament and of The Council of 9 March 2016 on Personal Protective Equipment and Repealing Council Directive 89/686/EEC.

[28] Asociación Española de Normalización (2005). UNE-EN−362: 2005, Personal Protective Equipment Against Falls from a Height. Connectors.

[29] Carrión E.A., Irles R., Segovia E.G., Pomares J.C. (2016). Personal fall arrest systems under impact. Numer. Simul. Inf. Constr..

[30] Goh Y.M., Love P.E.D. (2010). Adequacy of personal fall arrest energy absorbers in relation to heavy workers. Saf. Sci..

[31] Pomares J.C., Carrión E.A., González A., Saez P.I. (2020). Optimization on personal fall arrest systems. Experimental dynamic studies on lanyard prototypes. Int. J. Environ. Res. Public Health.

[32] Asociación Española de Normalización (1993). UNE-EN−364: 1993, Personal Protective Equipment against Falls from a Height. Test Methods.

[33] HBM Test and Measurement Messtechnik. HBM. Hottinger Brüel & Kjaer Ibérica, S.L.U.. www.hbm.com.

[34] Machines S.T. Servosis. www.servosis.com.

[35] Arteau J., Giguere D., Sulowski A.C. (1991). Proposed method to test harness for strength and human factors criteria. Fundamentals of Fall Protection.

[36] González A.M.N., Cobo A., Castaño A., Prieto M.I. (2015). A comparison of the resistance of Temporary Edge Protection Systems to static and dynamic loads. Inf. Constr..

[37] Irles R., Pomares J.C., Segovia E.G., Ferrer M.B., Carrión E.A., Schleyer G., Brebbia C.A. (2014). Soft retention in height fall safety devices. Proceedings of the 13th International Conference on Structures Under Shock and Impact.

[38] Kruskal W.H., Wallis W.A. (1952). Use of ranks in one-criterion variance analysis. J. Am. Stat. Assoc..

[39] Alcaraz J., Anton-Sanchez L., Monge J. (2020). ConcordanceTest: An Alternative to the Kruskal-Wallis Test Based on the Kendall Tau Ideas. R Package. https://CRAN.R-project.org/package=ConcordanceTest.

[40] Wilcoxon F. (1945). Individual comparisons by ranking methods. Biometrics.

[41] Standars Council of Canada, Conseil Canadien des norms (2015). CSA Z259.16−15 Design of Active Fall-Protection Systems.

[42] The American Society of Safety Engineers (2016). ANSI/ASSP Z359.6 Specifications and Design Requirements for Active Fall Protection Systems.

